# Past climate changes facilitated homoploid speciation in three mountain spiny fescues (*Festuca*, Poaceae)

**DOI:** 10.1038/srep36283

**Published:** 2016-11-03

**Authors:** I. Marques, D. Draper, M. L. López-Herranz, T. Garnatje, J. G. Segarra-Moragues, P. Catalán

**Affiliations:** 1 Departamento de Ciencias Agrarias y del Medio Natural, Escuela Politécnica Superior de Huesca, Universidad de Zaragoza, C/Carretera de Cuarte Km 1, E22071 Huesca, Spain; 2 Centro de Ecologia, Evolução e Alterações Ambientais (CE3C - Centre for Ecology, Evolution and Environmental Changes), C2, Universidade de Lisboa, Campo Grande, 1749-016 Lisbon, Portugal; 3 Institut Botànic de Barcelona (IBB-CSIC-ICUB), Passeig del Migdia s/n, 08038 Barcelona, Spain; 4 Departamento de Biología Vegetal, Facultad de Ciencias Biológicas, Universitat de València, Avda. Dr. Moliner, 50, E-46100, Burjassot, Spain; 5 Department of Botany, Institute of Biology, Tomsk State University, Lenin Av. 36, 634050 Tomsk, Russia

## Abstract

Apart from the overwhelming cases of allopolyploidization, the impact of speciation through homoploid hybridization is becoming more relevant than previously thought. Much less is known, however, about the impact of climate changes as a driven factor of speciation. To investigate these issues, we selected *Festuca picoeuropeana*, an hypothetical natural hybrid between the diploid species *F. eskia* and *F. gautieri* that occurs in two different mountain ranges (Cantabrian Mountains and Pyrenees) separated by more than 400 km. To unravel the outcomes of this mode of speciation and the impact of climate during speciation we used a multidisciplinary approach combining genome size and chromosome counts, data from an extensive nuclear genotypic analysis, plastid sequences and ecological niche models (ENM). Our results show that the same homoploid hybrid was originated independently in the two mountain ranges, being currently isolated from both parents and producing viable seeds. Parental species had the opportunity to contact as early as 21000 years ago although niche divergence occurs nowadays as result of a climate-driven shift. A high degree of niche divergence was observed between the hybrid and its parents and no recent introgression or backcrossed hybrids were detected, supporting the current presence of reproductive isolation barriers between these species.

Homoploid hybrid speciation (HHS), where interspecific gene flow leads to the formation of a novel and stable lineage without a change in chromosome number^1^,^2^,^3^ has traditionally been defended as rare in relation to the more common allopolyploid hybrid speciation^4^,^5^. But perspectives on the importance of homoploid speciation have drastically changed in the past decades since it has been documented in a wide number of plants and metazoan taxa^6^,^7^. However, to be evolutionarily successful, newly formed hybrid lineages need to overcome many negatively affecting factors such as reduced fertility and viability, lack of mates and meiotic abnormalities^8^,^9^. Even fertile hybrids face a major problem: to fully become a new species, the hybrid needs to establish self-sustaining populations that overcome competition with fitted parental species and genetic blurring through backcrossing and introgression^10^. Reproductive isolation between the hybrid and the parental species is therefore a key factor in the process of speciation, especially in homoploid hybrids that might easily be swamped by its established relatives.

Without changes in chromosome numbers, the isolation of the homoploid hybrid from the parental species could be facilitated by niche shifts resulting from new species tolerances^11^. Shifting climates can drastically change pre- and post-reproductive barriers such as spatial and temporal isolation or reproductive behavior which are critical in maintaining genetically pure species^12^,^13^ but they might also create new ecological niches where the hybrid might be more suitable than its parental species. Understanding how climate changes affect hybridization should therefore be a major question for predicting patterns of extinction and speciation.

In this study, we assessed how climate has shaped the outcomes of hybridization in two species of spiny fescues (*Festuca* L., Poaceae) where homoploid speciation was proposed to occur^14^,^15^. The spiny fescue *F. picoeuropeana* Nava (2*n* = 2*x* = 14) was suggested to be of hybrid origin because of its intermediate morphology between its putative parents, *F. eskia* Ramond *ex* DC. (2*n* = 2*x* = 14) and *F. gautieri* (Hack.) K. Richt. (2*n* = 2*x* = 14). The latter two species co-occur in several localities in the NE of Spain (Pyrenees) where they have presumably given rise to this hybrid^14^,^16^ although they also spread in allopatry in other regions of the Iberian Peninsula (NW Cantabrian Mountains, *F. eskia*; E Iberian Mountains, *F. gautieri*^15^). However, there are several allopatric hybrid populations in NW of Spain (Cantabrian Mountains; Fig. 1) located more than 400 km apart from the NE populations, where hybrid plants occur alone raising doubts about the origin of *F. picoeuropeana*.

Based on an extensive survey across the geographical range of these three fescues, we assessed the homoploid hybrid origin of *F. picoeuropeana.* A multidisciplinary approach was used combining data from chromosome counts, genome size estimation, chloroplast (cpDNA sequences), nuclear data (SSRs), seed viability and ecological niche modeling to specifically assess: (1) the homoploid hybrid nature of *F. picoeuropeana* in all populations; (2) evidence of hybridization in the genome of *F. picoeuropeana* and introgression with parental species; (3) isolation of the hybrid from the parental species; (4) fertility of the hybrid; and (5) whether climate-driven shifts in species distribution facilitated isolation between the hybrid and the parental species.

## Results

### Genome size and chromosome counts

Mean values for nuclear DNA amount were 5.70 ± 0.17 pg for *F. eskia*, 5.61 ± 0.20 pg for *F. gautieri* and 5.61 ± 0.20 pg for *F. picoeuropeana*. Differences in nuclear DNA content between these species were not statistically significant (Kruskall-Wallis test: *H* = 0.786 *P* = 0.675). All studied populations consistently yielded 2*n* = 14 chromosomes for *F. eskia*, *F. gautieri* and *F. picoeuropeana*, as reported previously (Table S1).

### Genetic composition of populations

Individuals morphologically assigned in the field either as *F. eskia* and *F. gautieri* retrieved high *q*-values both in STRUCTURE and NEWHYBRID analyses, either in allopatric or in sympatric populations where more than one species co-occurred (Fig. 2). By contrast, all individuals morphologically assigned as *F. picoeuropeana* showed intermediate *q*-values, at the best assignment result given by STRUCTURE (*K* = 2; Fig. 2A–C; Table S2). When STRUCTURE was run at higher *K* values, two geographically distinct clusters were identified for the samples of *F. picoeuropeana* showing a clear boundary between NW (Cantabrian Mountains) and NE (Pyrenees) hybrid populations (Fig. S1). STRUCTURE detected a further genetic substructure with an optimal *K* = 3 when all the allopatric *F. eskia* and *F. gautieri* samples were analyzed independently, grouping the southern Spain (Baetic Mountains), Cantabrian Mountains and Pyrenees populations of *F. gautieri* and the NE Cantabrian, NW Cantabrian and Pyrenees populations of *F. eskia* into different clusters (Fig. S2_1–2_). The same geographical results were obtained from BAPS using both the spatial (results not shown) and the non-spatial model (Fig. S2).

From the 419 individuals that could not be assigned confidently to the two pure parental species in STRUCTURE, a high proportion was assigned by NEWHYBRIDS as late-generation hybrids versus only 26% of F_1_ hybrids (Fig. 2; Table S2). Late-generation hybrids (F_2_-F_10_) were predominant in the majority of the populations, including 19 out of the 30 populations composed exclusively of *F. picoeuropeana* individuals (Fig. 2; Table S2). No recent backcrosses with the parental species were detected in any of the populations studied.

The PCoA analysis revealed two different isolated groups of samples, one formed by *F. eskia* and the other formed by *F. gautieri* in the opposite placements of the space defined by the two axes of the plot, which accounted for 24.63% of the variance (Fig. 3). F_1_ hybrids showed intermediate positions between the two progenitors independently of their geographical origin although most of the allopatric Cantabrian hybrids usually clustered in the lower left quadrant of the PCoA (Fig. 3). By contrast, late-generation hybrids were widely distributed between the parental species although two isolated clusters could be identified in the upper and lower levels of the PCoA (Fig. 3).

The NJ tree supported a separate origin of the hybrid in the two different geographical mountains (Fig. 4). Hybrids from the Cantabrian Mountains and Pyrenees populations always grouped with the parental species from their respective areas, even in the case of the allopatric hybrid populations occurring in the Cantabrian Mountains (Fig. 4).

### Genetic diversity

As expected in late generation hybrids, a very low number of the SSR alleles retrieved in *F. picoeuropeana* were inherited from both parents (8%). The remaining ones came from *F. gautieri* (42%) or from *F. eskia* (39%), although 11% of the alleles found in *F. picoeuropeana* were private alleles (Table S3). The comparative analysis of genetic diversity indices revealed that *F. gautieri* had significantly higher allelic richness and average observed heterozygosity and genetic diversity within populations than *F. eskia* (Table 1). Significant differences in these indices vanished when only allopatric populations of *F. eskia* and *F. gautieri* were considered (Table 1). In contrast, *F. picoeuropeana* showed significantly lower values of all genetic diversity indices except for observed heterozygosity when compared to *F. eskia* and *F. gautieri*. Allelic richness of *F. picoeuropeana* was similar in the two geographical areas.

### Genetic differentiation

The analysis of molecular variance indicated that 6.86%, 34.24% and 58.90% of the genetic variation in *F. picoeuropeana* was attributed to variations among the two clusters (Cantabrian Mountains and Pyrenees), among populations within clusters and within populations, respectively. Likewise, 4.13%, 7.68% and 88.19% of the genetic variation in *F. eskia* was attributed to variations among the two clusters, among populations within clusters and within populations, respectively. A similar trend was observed in *F. gautieri* (3.74%, 19.41% and 76.85%). A significant genetic differentiation was found between the two clusters of *F. picoeuropeana* identified using STRUCTURE (*F*_CT_ = 0.053 *P* > 0.00001). Nevertheless, the level of population structure was similar and non-significant within the two geographical clusters (Cantabrian Mountains: *F*_ST_ = 0.0047 *P* < 0.845; Pyrenees: *F*_ST_ = 0.0045 *P* < 0.982).

### Plastid DNA sequences

Statistical parsimony analyses based on the aligned matrix of the two plastid DNA regions (1520 bp long) yield one single network containing 33 haplotypes (Fig. 5). As expected from late generation hybrids, only a low number of the haplotypes found in *F. picoeuropeana* were shared with the parental species: two were shared with *F. eskia* and *F. gautieri* (II and IX), one with *F. gautieri* (XXIII). Six out of the ten haplotypes found in *F. picoeuropeana* occurred exclusively in the Cantabrian Mountains (XXIII, XXXIX to XXXIII), three (IX, XXVII and XXVIII) in the Pyrenees and one (II) was shared between both mountain ranges (Fig. 5). The number of haplotypes was lower in *F. eskia* but higher in *F. gautieri* (9 and 21 haplotypes, respectively). This latter species also yielded the greatest nucleotide diversity (*π*: *F. gautieri*: 0.00201; *F. eskia*: 0.00158; *F. picoeuropeana*: 0.00140). As expected, all F_1_ hybrids had the same chloroplast sequence as their maternal progenitor while most late-generation hybrids usually had different chloroplast sequences (Fig. 5).

### Ecological niche modeling

**Figure 1:**
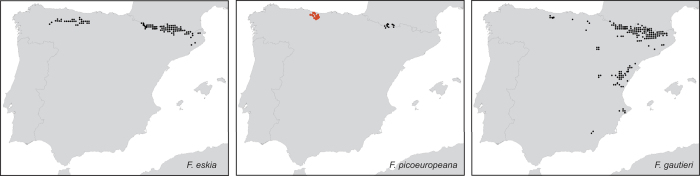
Current known distribution of *Festuca eskia*, *F. gautieri* and the hybrid *F. picoeuropeana* (allopatric hybrid populations are indicated in red). Maps were generated with Idrisi Selva v.17.02 environment (Clark Labs, Clark University, www.clarklabs.org).

Present models were consistent with their known distribution (Fig. 1) although they support a large reduction and a shift in the area suitable for the occurrence of *F. eskia* and *F. gautieri* since the LGM (*D*_LGM_ = 0.4972) to our current days (*D* = 0.2186). Past ENMs suggested that *F. eskia* and *F. gautieri* had the opportunity for contact as early as the LGM, both in the Cantabrian Mountains and the Pyrenees (Fig. 6A,B; for more detail see Fig. S3_1–2_). The area where these two species potentially co-existed was much wider in the past with an overlap in the areas where *F. picoeuropeana* currently occurs without *F. eskia* and *F. gautieri* (Fig. 6A,B; Fig. S4_1–2_).

The niches of *Festuca eskia* and *F. picoeuropeana* populations showed a higher overlap in the LGM than at current conditions and a parallel niche overlap regression from LGM to current conditions was observed in these two species (Table S4). A high reduction in niche overlap occurred in the Cantabrian Mountains and the Pyrenees populations of *F. picoeuropeana* between both periods, decreasing from *D*_LGM_ = 0.3461 to just *D* = 0.0721 at current conditions (Table S4).

The ecological conditions of the hybrid populations occurring in the Cantabrian Mountains and in the Pyrenees are significantly different since the ENMs of the Cantabrian populations do not predict their occurrence in the Pyrenees and *vice versa* (Fig. 6; for more details see Fig. S4_1–2_). The LGM projections of the Cantabrian populations revealed a migration to the South, where the current allopatric populations exist, while the LGM projections of the Pyrenean populations revealed the disappearance of suitable conditions in the East of the Pyrenees (Fig. 6C,D). Niche similarity tests also indicated statistically significant differences between the two geographic groups of *F. picoeuropeana* (Table S4). The tests of current niche divergence and conservatism between each pairwise comparison supported niche divergence between all pairs of species with the exception of the Pyrenean group of *F. picoeuropeana* with both parental species (*F. eskia* and *F. gautieri*) that showed niche conservatism (Fig. 7).

### Seed viability of parental and hybrid species

Germination percentages showed an average of 55.8 ± 3.51% in *F. gautieri*, 81.50 ± 9.61% in *F. picoeuropeana*, and 91.5 ± 0.82% in *F. eskia*. Results indicated that the hybrid produced fertile seeds that showed similar germination percentages to those of *F. eskia* (*t* = 1.970, *P* = 0.096) and outperformed those of *F. gautieri* (*t* = 5.669, *P* = 0.0001; Fig. S5). No differences in seed germination were found between sympatric and allopatric hybrid populations (*t* = 25.092, *P* = 0.891).

## Discussion

### Homoploid hybrid speciation facilitated by climate-driven shifts

During homoploid hybrid speciation, a new fertile hybrid lineage is expected to arise through the establishment of long-lasting populations, reproductively isolated from the parental species, to escape the homogenizing effects of gene flow^17^. Here, we showed that homoploid speciation has occurred in two different geographical mountain areas within the studied group of *Festuca*. As expected under this hypothesis, we found the same ploidy level among species and molecular results sustained the hybrid origin of *F. picoeuropeana*. We found late generation hybrids in all populations, although predominant in the Cantabrian populations where parental species are no longer present. Few plastid haplotypes were shared with the parental species and few SSR alleles came from both parental species, which would be the case if hybrids were from early generations.

The high seed germination found in the hybrids, with the same or even higher germinability than the seeds from their parental species confirmed that *F. picoeuropeana* is able to produce fertile seeds and establish self-sustaining populations. There are numerous examples reporting hybrids that are as successful as their parental species^11^,^18^, in some cases even outcompeting them^19^. Despite we have no data concerning the compatibility system of these fescues, they show grass features compatible with allogamy^20^ like those reported in many other fescues^14^. But the three species studied here are also pseudorrhizomatous plants, spreading primarily by asexual reproduction^15^, which can contribute to the persistence of their populations^19^. It implies that any hybrid generation could last over many years in the same area without any crossing, favoring its geographical isolation.

### Factors responsible for isolation between hybrids and parental species

A crucial question in speciation is the confirmation that the homoploid hybrid is reproductively isolated from its parents. Although we have not directly tested the levels of pre- and post-reproductive barriers, the majority of hybrid populations occur in areas where the parental species are not present, which implies a major geographical isolation barrier. Our LGM projections revealed that the area favorable for the occurrence of these fescues was much wider in the past but subsequent climate changes have induced a loss of suitable habitat, forcing the species to shift their ranges to more divergent ecological areas, limiting the areas of contact and of potential hybridization. The occurrence of common niches where both parental species could have co-occurred and lead to hybridization was more widespread in the past suggesting that niche isolation between these species evolved across time and that currently constitutes an important barrier against introgression.

The colonization of new ecological niches was probably an important step for the speciation of *F. picoeuropeana*. Within each mountain area, habitat contractions were followed by colonization of new sites since the ENMs showed that the hybrid migrated to the South in the Cantabrian Mountains and to the West in the Pyrenees. All these processes of contraction and expansion in species distributions have the potential to promote periods of isolation and secondary contact of populations, fostering speciation^21^ and provide an explanation for the origin of this natural hybrid. Climatic changes can also facilitate the displacement of parental species if the new hybrid is more fitted than the parents to the new ecological changes^22^.

However, other isolation barriers should be currently acting to facilitate the isolation of the hybrid from the parental species, especially in the areas where the three species co-exist. The fact that *F. eskia* and *F. gautieri* have been shown to retain their genetic integrity (i.e. not showing genetic admixture or backcrossed genotypes), supports the existence of local barriers against interspecific gene flow. Phenological asynchrony, one of the most effective barriers to hybridization in several plant studies^17^, could also restrain gene flow between *F. eskia* and *F. gautieri*. Despite the fact that these two fescues bloom usually in July, co-blooming populations may still achieve a high degree of isolation if anthesis is asynchronous. This pattern has been reported in other fescues^23^ and might also act as a barrier to hybridization in the fescues studied here. Asexual reproduction through pseudorrhizomes should also be considered a major isolation barrier between these fescues since seedlings are infrequent^15^.

### Did speciation occur independently in two different mountain systems?

The geographic partitioning of genetic structure retrieved from hybrid populations suggests that two speciation events could be postulated in our study. The fact that hybrid populations in the Cantabrian Mountains have different chloroplast haplotypes than the ones in Pyrenees and the geographical partitioning of SSR genetic structure in two main groups is consistent with the hypothesis that the hybrid arose independently in these separate mountains, where populations occur more than 400 km apart. The correlated geographical and genetic boundaries support the fact that genome composition of the hybrids varies geographically which means that it was generated mainly by neighboring parental species. A similar pattern has been observed in *Argyranthemum sundingii* where multiple origins were proposed^24^. It is true that hypothesis of dispersal *vs.* multiple origins should be considered carefully when considering cases of hybridization since geographical patterns of molecular variation can have alternative explanations like a single origin followed by dispersal of ancestral genetic variation^11^. Nonetheless, ENMs supports the idea of two independent origins for *F. picoeuropeana* since they showed the existence of divergent climatic requirements in the Cantabrian and Pyrenean populations and a low degree of Schoener’s D niche overlap (0.07; Table S4). We are aware that these low values of niche overlap must be taken with extreme caution since we are comparing species with very different distribution ranges, which poses problems of scale^25^. ENMs based only on climatic data in narrow distributed species are indeed susceptible to spatial autocorrelation^25^ but climatic differences between geographically isolated populations have been found in other cases^26^. Further, we would expect a significant reduction in genetic diversity after a long-distance dispersal event. However, genetic diversity showed similar levels in both geographic regions supporting the hypothesis of independent origins for *F. picoeuropeana*.

## Conclusions

Mountains provide a natural laboratory to examine the impacts of climate change upon species distributions. Glacial cycles have often been invoked to explain distributional shifts of species, as well as contraction, fragmentation or connectivity of mountain populations^21^. Such dynamic systems, where both isolation and connectedness are possible, together with the possibility of different ecotones, provide an excellent cradle for speciation to occur. Despite many studies predict that climate changes might facilitate hybridization between previously isolated lineages in newly arisen contact zones, our study supports a contrasting scenario where the origin of the same new homoploid hybrid was facilitated by climate changes. The changes induced by the climate in the distributional patterns of the parental species of these fescues have restrained their current areas of contact, limiting the potential areas of hybridization between *F. eskia* and *F. gautieri*. It has also triggered the migration of the hybrid into new areas, generating a geographical barrier between the hybrid and the parental species, which facilitated the process of hybrid isolation and ultimately the speciation of *F. picoeuropeana*.

## Material and Methods

**Figure 2:**
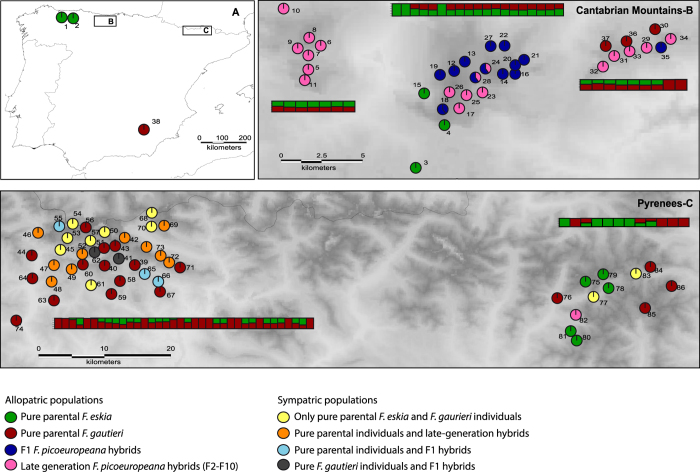
Overall distribution of the populations sampled for the three fescues and their assignments to genetic clusters. An elevation layer has been included in maps B and C to show the spatial isolation between populations. (**A**) Allopatric populations (1, 2 and 38) sampled outside the two major mountain ranges studied here (**B,C**). (**B**) Populations sampled in the Cantabrian Mountains. (**C**) Populations sampled in Pyrenees. Bars indicate posterior probabilities (*q*) for each population analyzed with STRUCTURE using the best assignment analysis (*K* = 2). Pie charts indicate posterior probabilities (*q*) for each population analyzed with NEWHYBRIDS. Colors indicate an assignment probability, according to different categories. Maps were generated with Idrisi Selva v.17.02 environment (Clark Labs, Clark University, www.clarklabs.org).

**Figure 3:**
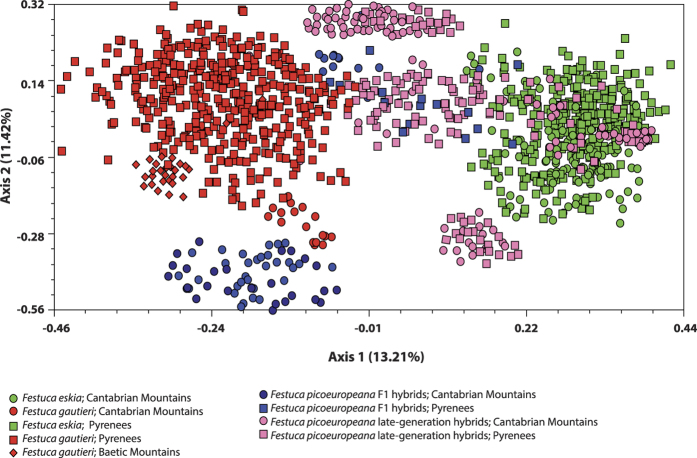
PCoA of mountain spiny fescues based on SSR markers and on the genotype assignment of NEWHYBRIDS. Species identities and geographic origins of populations are indicated in the figure.

**Figure 4:**
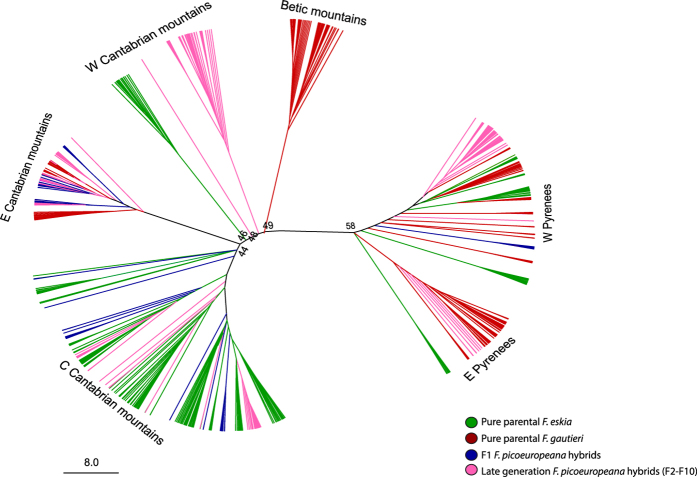
Neighbour-joining tree based on Nei’s unbiased genetic distance (Da). Monophyletic branches from the same population were collapsed except if individuals had a different genetic assignation. Only BS higher than 40% are shown. Color codes for taxa and for hybrid generation types are indicated in the figure.

**Figure 5:**
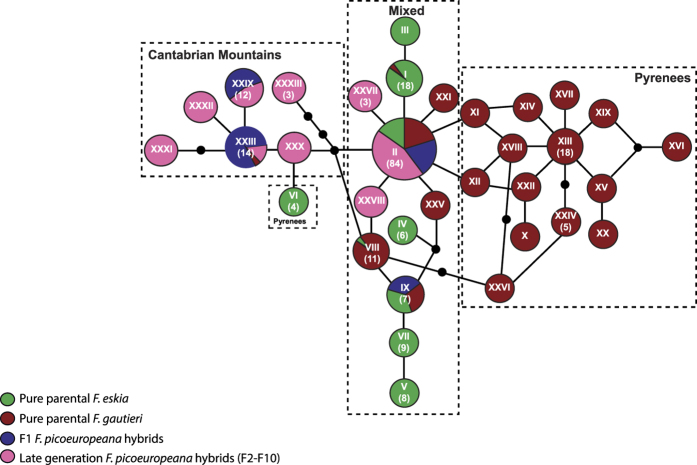
Statistical parsimony plastid network of the three studied fescues. Network pie charts indicate the percentage of sequences of each taxon and type of hybrid generation in each haplotype. Color codes for taxa and for hybrid generation are indicated in the lower left corner.

**Figure 6:**
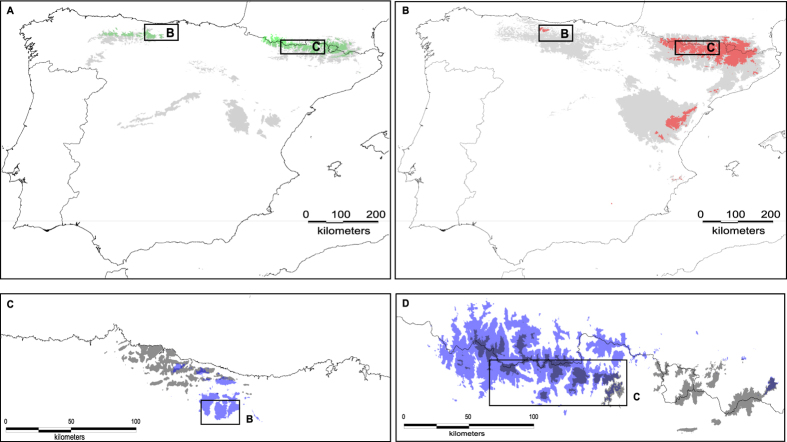
Current ENMs of *F. eskia* (green) and *F. gautieri* (red), *F. picoeuropeana* (blue), and in their respective LGM-CCSM scenarios (grey), based on the Maxent algorithm. Absence of colors indicates no favorable ecological conditions for the occurrence of the species. (**B,C**) Squares indicate the two major mountain ranges where current populations where sampled as indicated in Fig. 2. (**A**) ENMs of *F. eskia*; (**B**) ENMs of *F. gautieri*; (**C**) ENMs of *F. picoeuropeana* using only the Cantabrian records; (**D**) ENMs of *F. picoeuropeana* using only the Pyrenean records. Maps were generated with Idrisi Selva v.17.02 environment (Clark Labs, Clark University, www.clarklabs.org).

**Figure 7:**
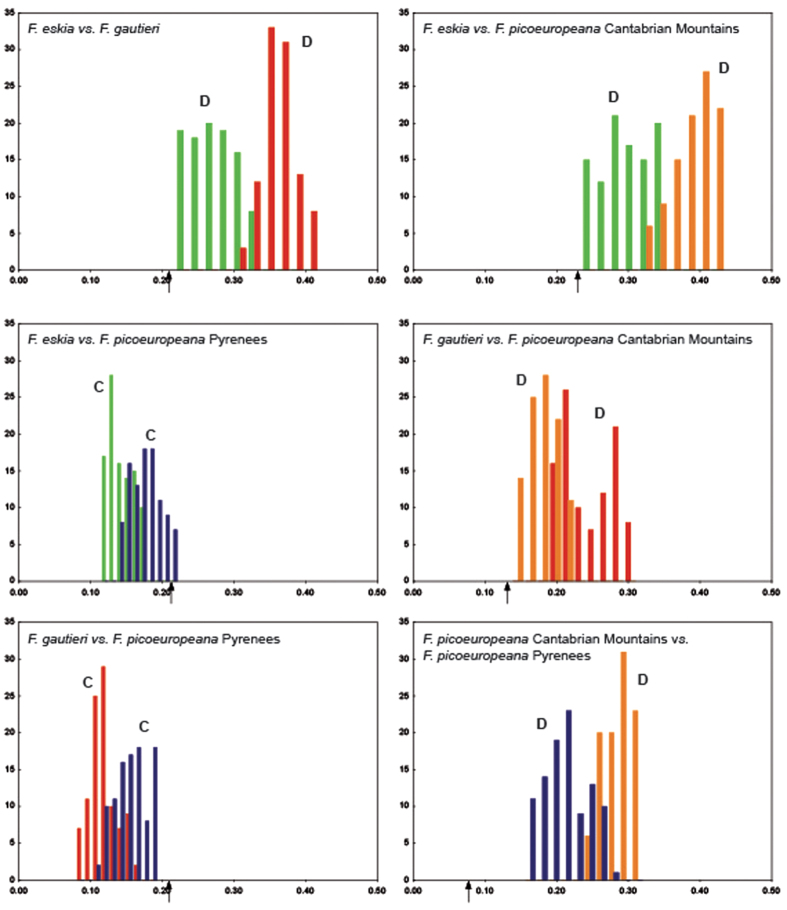
Pairwise comparison between the two parental species and the two populations of *F. picoeuropeana* to test niche divergence and conservatism under current climatic conditions. Schoener’s D niche-overlap values (arrows) are compared to a null distribution of background divergence. Values smaller than the null distribution suggest niche divergence (**D**), whereas larger values indicate niche conservatism (**C**). Key colors: *F. eskia* (green); *F.gautieri* (red); Cantabrian *F. picoeuropeana* (orange); Pyrenean *F. picoeuropeana* (blue).

In total, 1213 individuals were sampled (385 *F. eskia*, 470 *F. gautieri* and 358 *F. picoeuropeana*), covering the entire distribution range of these species in a total of 86 localities (Fig. 2, Table S1). No other co-blooming fescues that could cross with these species occurred in the study area. Species are perennial rhizomatose grasses with a generation time of two or more years, reproducing very frequently by asexual reproduction (pseudorrhizomes). Species identity was determined following the criteria of^15^. Individuals were collected randomly with a minimum sampling distance of 10 m to avoid the sampling of relatives or ramets. Fresh leaves were collected for each individual, dried in silica gel and stored at –20 °C until ready for DNA isolation. Genomic DNA was extracted using the DNeasy Plant Minikit (QIAGEN, Barcelona, Spain) following the manufacturer’s instructions. A detailed description of the following methods is provided in the Expanded Methods Section online (File S1).

### Ploidy level and chromosome counts

Fresh young leaves of the plants were co-chopped using a razor blade with an internal standard in the proportions 2:1 in 1200 μl of LB01 buffer^27^ with 0.5% Triton X-100 and supplemented with 100 μg/ml ribonuclease A (RNase A, Boehringer, Meylan, France) in a plastic Petri dish. For each population analyzed (Table S1) two samples of each individual collected were extracted and measured independently. Fluorescence analysis was carried out using an Epics XL flow cytometer (Coulter Corporation, Hialeah, Florida, USA) at the Centres Científics i Tecnològics de la Universitat de Barcelona (Spain) with the standard configuration as described in ref. 28.

### Molecular studies

Ten previously developed nuclear microsatellite loci were used to genotype all 1213 samples following the amplification protocols described in ref. 29. The products were run on an ABI 3730 automated sequencer (Applied Biosystems, Madrid, Spain) using LIZ500 as the internal lane size standard, and fragments were assigned to allele classes using Genemarker v. 1.85 software (Softgenetics, State College, PA, USA). Allelic richness (A*), observed heterozygosity (*H*_O_), within populations genetic diversity (*H*_S_), inbreeding coefficient (*F*_IS_) and population differentiation (*F*_ST_) were estimated with FSTAT v. 2.9.3.2^30^, and differences were tested for significance with 1000-permutation tests.

STRUCTURE version 2.2^31^ was first run from *K* = 1 to *K* = 40 to identify the best *K* value using all samples. A second STRUCTURE analysis was performed independently for each of the three species. All analyses were based on an admixture ancestry model with correlated allele frequencies and performed with a burn-in period and a run length of the Monte Carlo Markov Chain (MCMC) of 5 × 10^4^ and 5 × 10^5^ iterations, respectively, and each run was replicated ten times. To confirm the consistency of our results, samples were also analyzed using a different algorithm, as implemented on BAPS 5.2^32^. Clustering analysis with BAPS was done at the level of groups of individuals independently using two models (with and without spatial information). Each analysis was done selecting 2–90 as *K* values with ten replicates for each *K* value.

NEWHYBRIDS version 1.1.beta^33^ was used to determine the type of hybrid generations with the following genetic classes: pure parental *F. eskia*, pure parental *F. gautieri*, backcross genotypes with *F. eskia* and with *F. gautieri*, F_1_ hybrids, and later generation hybrids (run from F_2_ to F_10_). Because the power of detection of late hybrid generations is limited^34^,^35^ we did not attempted to distinguish those different classes and treated them in this study as a single group of late-hybrids^36^. A considerable amount of genetic data is required to distinguish genealogical classes and it is very difficult to accurately resolve further genealogical classes or second-backcrossed hybrids with NEWHYBRIDS or any other Bayesian Program^37^. NEWHYBRIDS analyses were based on the same computational parameters as those conducted in STRUCTURE using a threshold of *q* = 0.90.

Nei’s unbiased genetic distance (Da) was calculated with GenAlEx6^38^ among all pairs of samples classified according to the genetic membership obtained from NEWHYBRIDS and visualized by Principal Coordinates Analysis (PCoA) using NTSYSpc 2.11^39^. The same matrix of genetic distances was also used to construct a Neighbour-Joining (NJ) tree in the same program with 500 bootstrap replications. Genetic differentiation was calculated using an analysis of molecular variance (AMOVA) with ARLEQUIN v. 3.11^40^. Variance components were calculated among groups, among locations within groups and within sampling locations. Each AMOVA was run with 10000 permutations at 0.05 significance level.

Plastid *trn*T*- trn*L and *trn*L*-trn*F DNA sequences were amplified using the primers of ref. 41 and the PCR conditions described in ref. 42 for 221 individuals (Table S1). Forward and reverse individual sequences were assembled using SEQUENCHER 4.1.4 (GeneCodes Corp., Ann Arbor, MI, USA) and corrected where necessary. Sequences were deposited in Genbank (Table S5). Within-population haplotype diversity (*π*) was estimated with DnaSP 5.10.1^43^. The two plastid regions were concatenated and analyzed using statistical parsimony as implemented in TCS^44^.

### Environmental niche modeling

To generate environmental niche models (ENMs), a total of 627 unique records were used considering a pixel resolution of approximately 1 km^2^. To test if the hybrid populations shared the same ecological niche, two “subpopulations” of *F. picoeuropeana* were considered and analyzed separately: the Cantabrian Mountains and the Pyrenees. Occurrence data were obtained from fieldwork, herbarium records and contrasted datasets such as the GBIF Database (data.gbif.org) and the SIVIM Database (www.sivim.info). GIS layers used in the ENMs included the 19 climate data variables from Worldclim^45^, (http://www.worldclim.org/) with a 30 arc-second resolution (approximately 1 km^2^) and the altitude obtained from SRMT (http://srtm.csi.cgiar.org/) all adjusted at the same spatial resolution. ENMs were generated using the Maxent algorithm^46^, which estimates a target probability distribution by finding the probability distribution of maximum entropy subject to a set of constraints that represent the incomplete information about the target distribution^47^. Model fitness exhibited high AUC values for the two parental species (*F. eskia*: 0.89; *F. gautieri*: 0.93) and also for the hybrid, *F. picoeuropeana* (0.79 and 0.81 for the Cantabrian Mountains and the Pyrenees, respectively).

A single background area was defined for all species, as well as a buffer area of 260 km following the optimization of the AUC criteria^48^. Variable selection was performed using a correlation analysis (*R* ≥ 0.75; Pearson coefficient) and only the most biologically relevant and non-correlated variables were retained for subsequent modeling (Table S6). Seventy-five percent of the occurrence locations were used for building the model, and the remaining 25% were used for testing the accuracy of the model. Past projections were based on the Last Glacial Maximum, LGM-CCSM circulation model^45^ and downloaded from http://www.worldclim.org/paleo-climate.

### Null models testing niche divergence versus conservatism in *Festuca*

To evaluate ecological niche overlap across the LGM and the current time, we used Schoener’s *D* and Hellinger’s-based *I* index included in ENMTools v1.4.4^49^,^50^. Both measures give a similarity value ranging from 0 to 1, with 0 indicating no niche overlap and 1 indicating completely similar niches. Similarity tests were performed to test if niche divergences have driven differentiation^25^ both between parental and hybrid species and between the parental species for the current time. Background selection followed the procedure used in ref. 25 and thresholds of ENM’s were selected using the minimum model value of current occurrence point^51^. For these tests, a null distribution is generated for the ENM difference between one species and a random sample of the background climate available to the other species^52^. These tests were also performed between the Cantabrian and Pyrenean populations of *F. picoeuropeana.*

### Seed viability of the two parental fescues and hybrid species

To compare seed viability between parental species and the hybrid, seeds were collected in five parental populations and six hybrid populations: three sympatric and three allopatric populations (Table S1). Four replicates of 25 seeds from each species and per population were sown in Petri dishes on moistened filter paper for 30 days. Previously, seeds had been imbedded for three weeks at 15 °C to break seed dormancy. After this moist stratification, seeds were incubated under darkness conditions in growth chambers with a constant temperature of 15 °C for *F. eskia* and *F. picoeuropeana*, and 20 °C for *F. gautieri*. Every two days, germinated seeds were counted and removed and, at the end of each assay, final germination percentages were assessed (average ± SD across replicates). Data were arcsine square root-transformed, and differences in seed germination between hybrid and parental species, and between sympatric and allopatric hybrid populations, were analyzed by a t-test using SPSS 11.0 (SPSS, Inc., Chicago, Illinois, USA).

## Additional Information

**How to cite this article**: Marques, I. *et al.* Past climate changes facilitated homoploid speciation in three mountain spiny fescues (*Festuca*, Poaceae). *Sci. Rep.* **6**, 36283; doi: 10.1038/srep36283 (2016).

**Publisher’s note:** Springer Nature remains neutral with regard to jurisdictional claims in published maps and institutional affiliations.

## Supplementary Material

Supplementary Information

Supplementary Table S1

Supplementary Table S2

Supplementary Table S3

Supplementary Table S4

Supplementary Table S5

Supplementary Table S6

## Figures and Tables

**Table 1:** Pairwise comparison of SSR average genetic diversity parameters between the three species (interspecific comparisons) of *Festuca* and between allopatric and sympatric populations of each species (intraspecific comparisons).

Groups/indices	*N*	A*	*H*_O_	*H*_S_	*F*_IS_	*F*_ST_
**Interspecific comparisons, all populations**						
*F. eskia*	34	1.653^a^	0.522^a^	0.635^a^	+0.178^a^	0.157^a^
*F. gautieri*	45	1.741^b^	0.602^b^	0.741^b^	+0.189^a^	0.129^a^
*F. picoeuropeana*	41	1.551^c^	0.513^a^	0.521^c^	+0.016^b^	0.367^b^
**Interspecific comparisons, allopatric populations**						
*F. eskia*	10	1.620^a^	0.460^a^	0.616^a^	+0.252^a^	0.160^a^
*F. gautieri*	20	1.728^a^	0.631^b^	0.724^a^	+0.129^a^	0.156^a^
*F. picoeuropeana*	27	1.458^b^	0.474^a^	0.409^b^	−0.158^b^	0.469^b^
**Intraspecific comparisons. allopatric** ***vs*****. sympatric populations**						
*F. eskia*	10	1.620^a^	0.461^a^	0.616^a^	+0.252^a^	0.160^a^
*F. eskia* sympatric only with *F. gautieri*	12	1.644^a^	0.509^a^	0.616^a^	+0.173^a^	0.162^a^
*F. eskia* sympatric with *F. gautieri* and *F. picoeuropeana*	12	1.678^a^	0.633^b^	0.684^a^	+0.075^a^	0.052^a^
*F. gautieri*	20	1.727^a^	0.631^a^	0.723^a^	+0.129^a^	0.156^a^
*F. gautieri* sympatric only with *F. eskia*	12	1.744^a^	0.559^a^	0.734^a^	+0.242^a^	0.124^a^
*F. gautieri* sympatric with *F. eskia* and *F. picoeuropeana*	12	1.758^a^	0.622^a^	0.786^a^	+0.209^a^	0.063^a^
*F. picoeuropeana*	27	1.458^a^	0.474^a^	0.409^a^	−0.158^a^	0.469^a^
*F. picoeuropeana* sympatric with *F. eskia* and *F. gautieri*	12	1.730^b^	0.603^b^	0.764^b^	0.211^b^	0.097^b^

A* = allelic richness;

*H*_O_, *H*_S_, average observed and expected heterozygosity within populations, respectively; F_IS_: inbreeding coefficient; F_ST_: coefficient of genetic differentiation. Different superscript letters for the compared index values indicate significant differences at *P* < 0.05.
