# Supplementary material for: Past climate changes facilitated homoploid speciation in three mountain spiny fescues (Festuca, Poaceae)
Source: Sci Rep. 2016 Nov 3;6:36283. doi: 10.1038/srep36283 (PMC5093761; doi:10.1038/srep36283)
Supplement: Supplementary Information [file srep36283-s1.pdf]

SUPPLEMENTARY INFORMATION

Past climate changes facilitated homoploid speciation in three mountain spiny fescues (*Festuca*, Poaceae)

Marques I, Draper D, López-Herranz ML, Garnatje T, Segarra-Moragues JG, Catalán P.

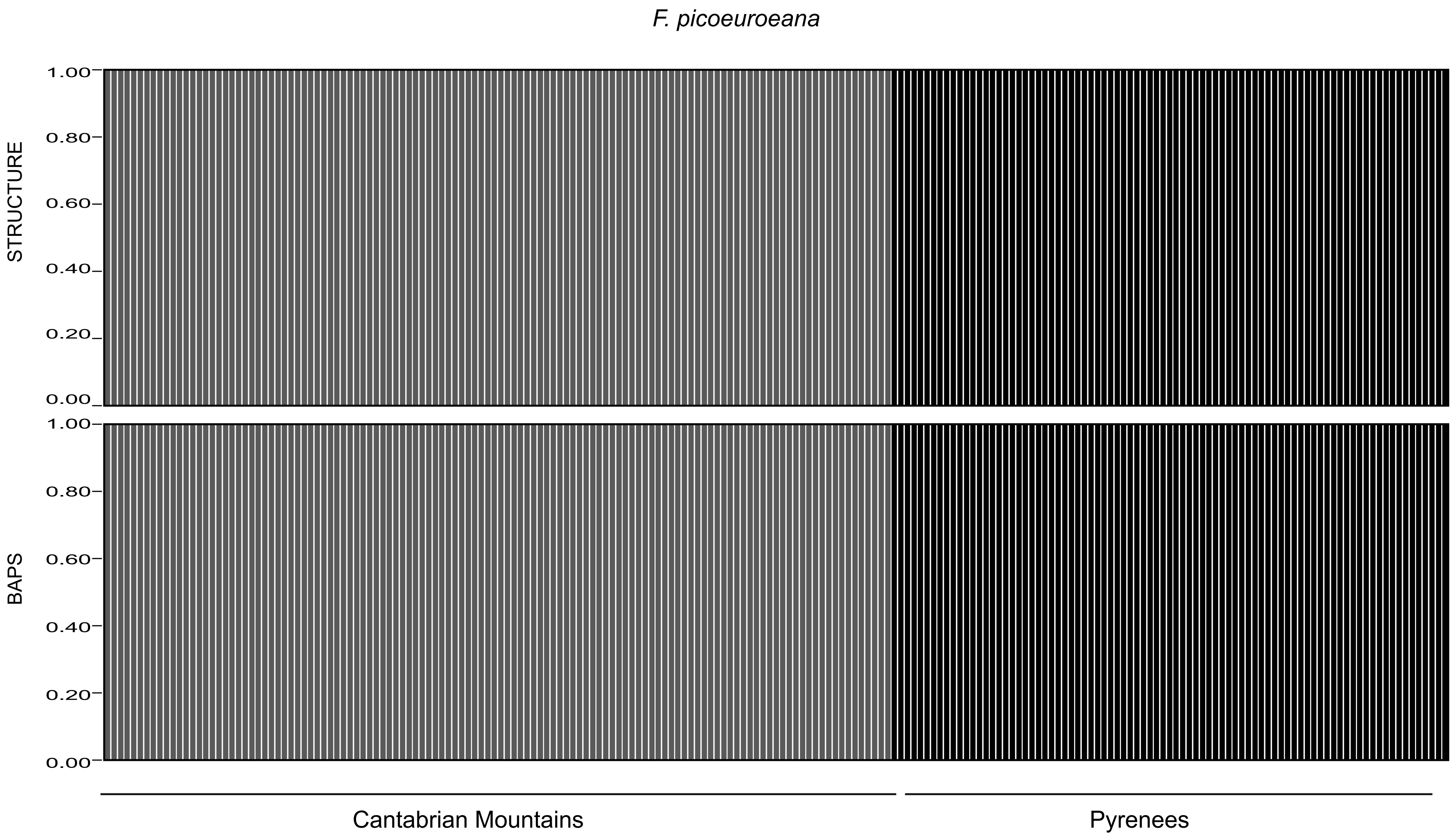

Figure S1. Posterior probabilities for each analyzed individual of Festuca picoeuropeana using the best assignment analysis of STRUCTURE (K = 2) run only with this species.

Past climate changes facilitated homoploid speciation in three mountain spiny fescues (Festuca, Poaceae)
Marques I, Draper D, López-Herranz ML, Garnatje T, Segarra-Moragues JG, Catalán P.

1)

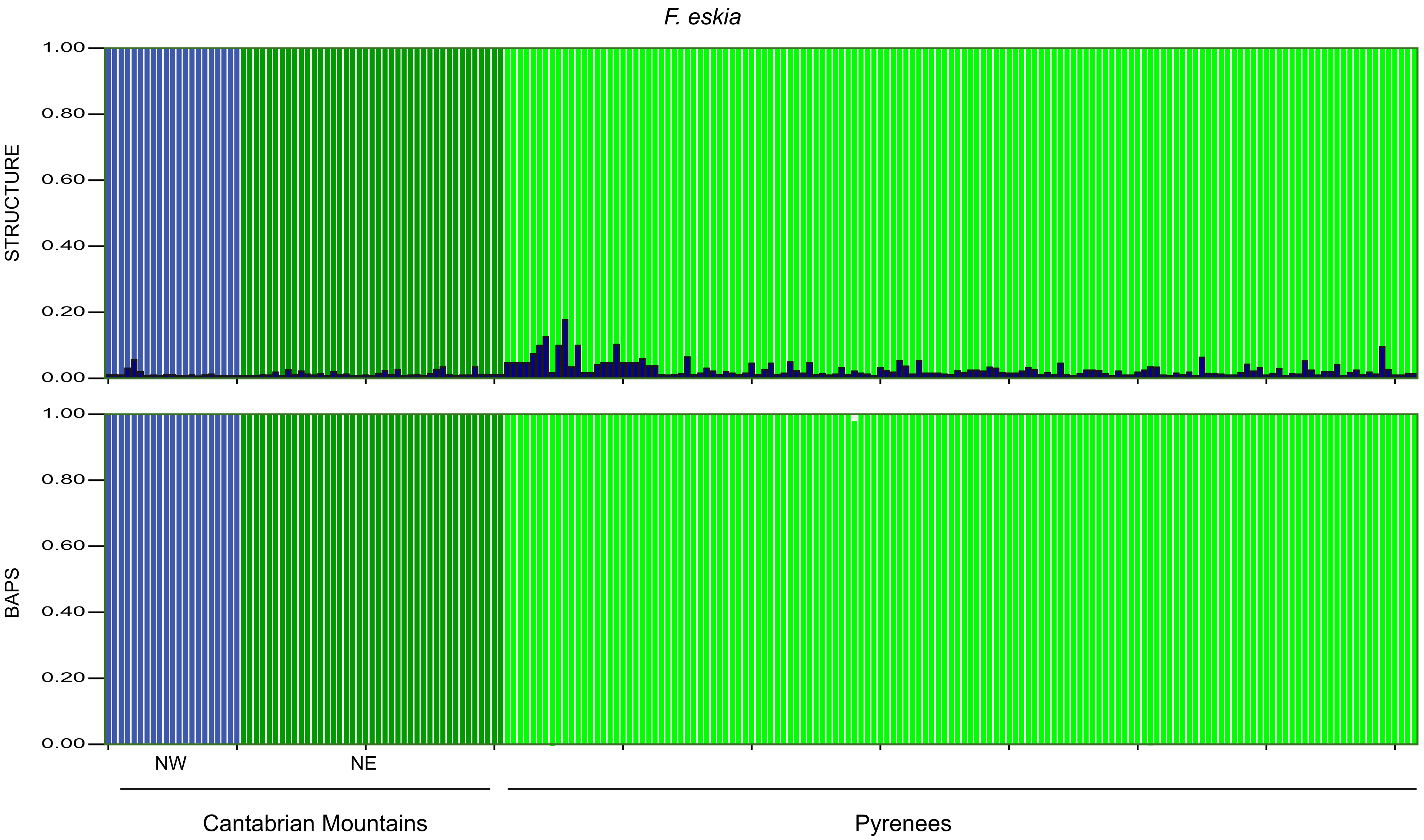

Figure S2. Posterior probabilities for each analyzed individual of *Festuca eskia* (1) and *F. gautieri* (2) using the best assignment analysis of STRUCTURE and BAPS (K = 3). Analyses were performed independently for each species.

Past climate changes facilitated homoploid speciation in three mountain spiny fescues (Festuca, Poaceae)
Marques I, Draper D, López-Herranz ML, Garnatje T, Segarra-Moragues JG, Catalán P.

2)

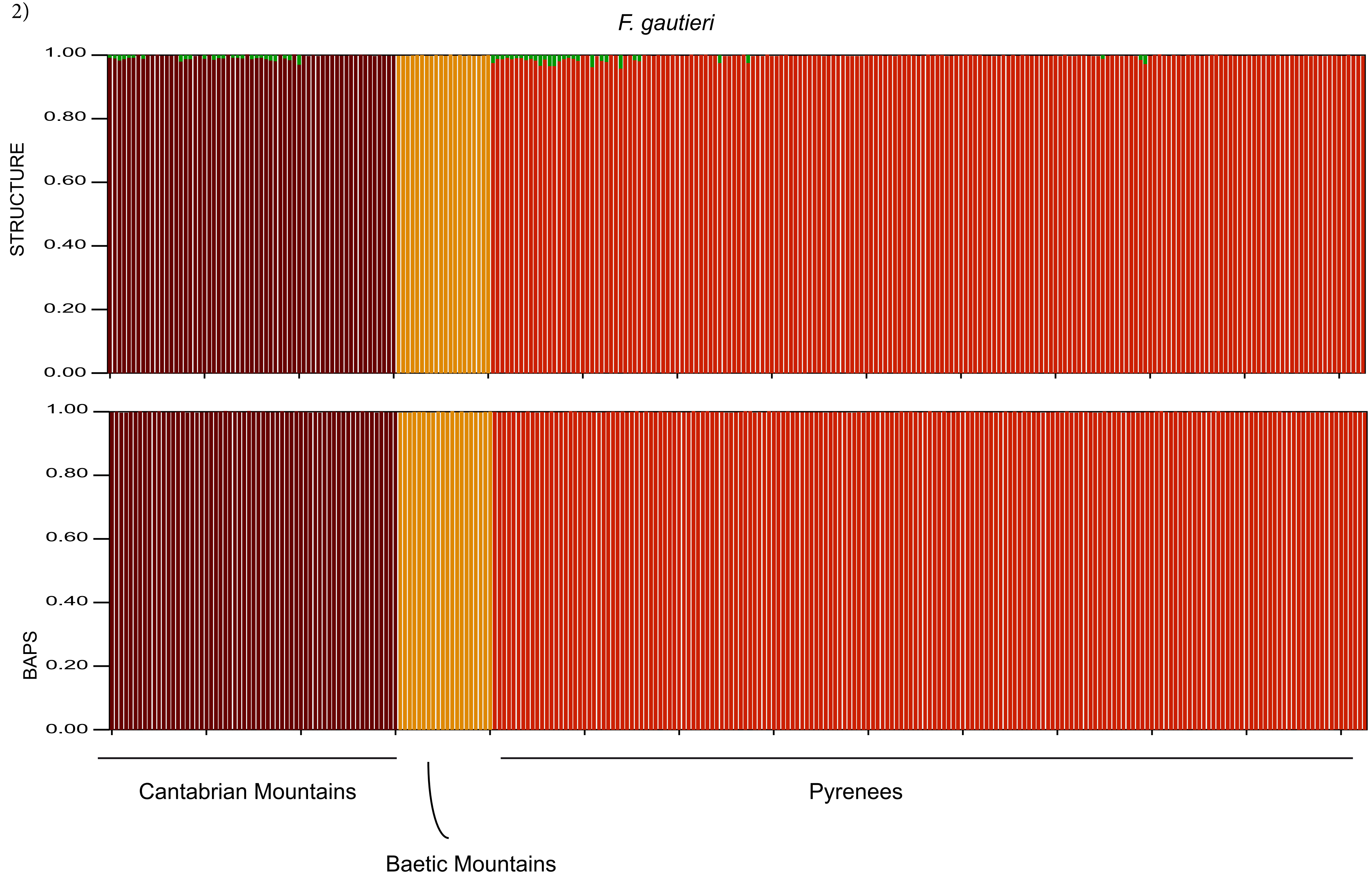

Figure S2. Posterior probabilities for each analyzed individual of *Festuca eskia* (1) and *F. gautieri* (2) using the best assignment analysis of STRUCTURE and BAPS (K = 3). Analyses were performed independently for each species.

Past climate changes facilitated homoploid speciation in three mountain spiny fescues (Festuca, Poaceae)
Marques I, Draper D, López-Herranz ML, Garnatje T, Segarra-Moragues JG, Catalán P.

1)

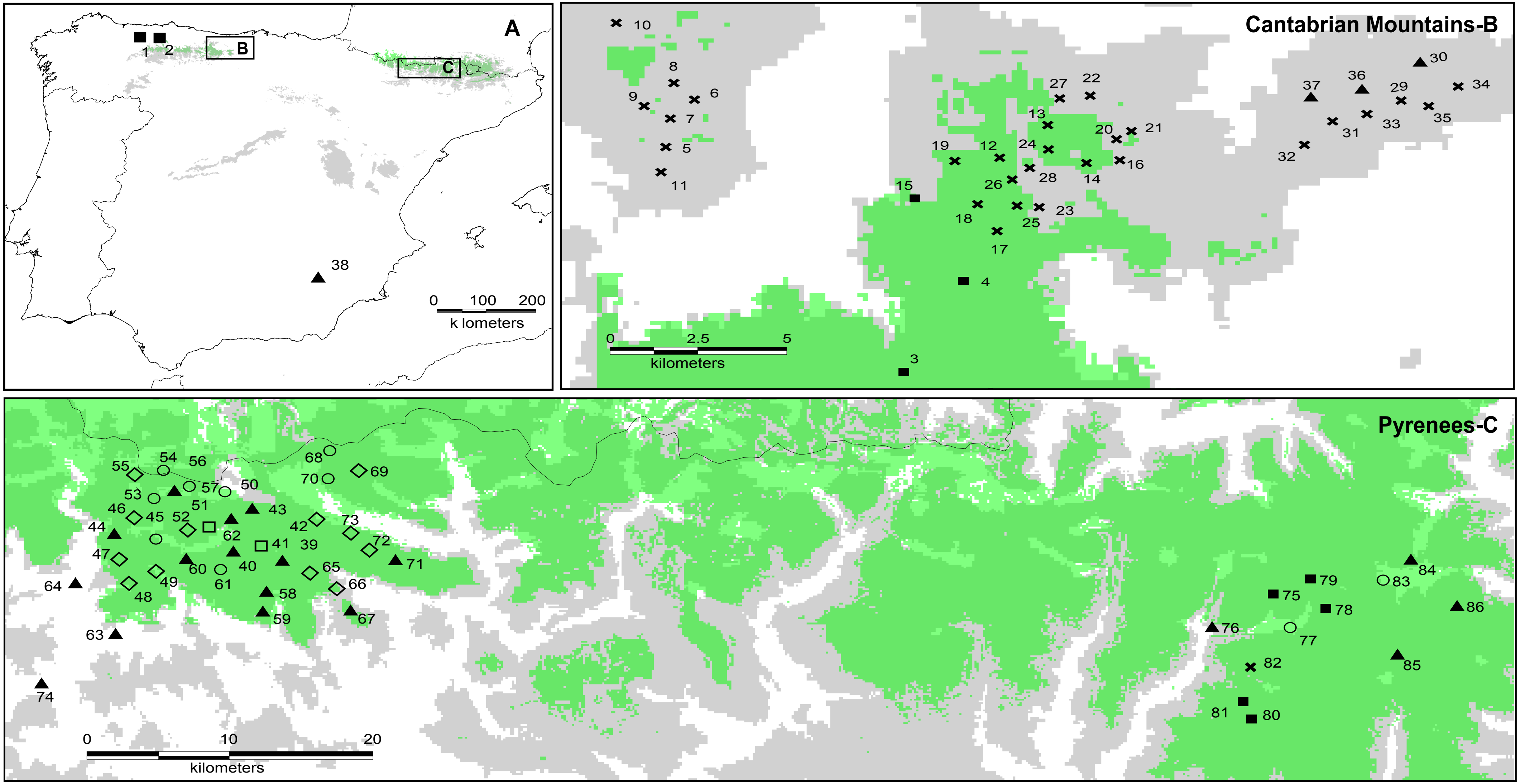

Figure S3. Ecological niche models of *F. eskia* (1) and *F. gautieri* (2) projected to LGM (grey) and to current (green for *F. eskia* and red for *F. gautieri*) climatic conditions. Predominance of one color indicates niche differentiation while dark color indicates overlapping of niche models. Maps were generated with Idrisi Selva v.17.02 environment (Clark Labs, Clark University, www.clarklabs.org).

Past climate changes facilitated homoploid speciation in three mountain spiny fescues (Festuca, Poaceae)
Marques I, Draper D, López-Herranz ML, Garnatje T, Segarra-Moragues JG, Catalán P.

2)

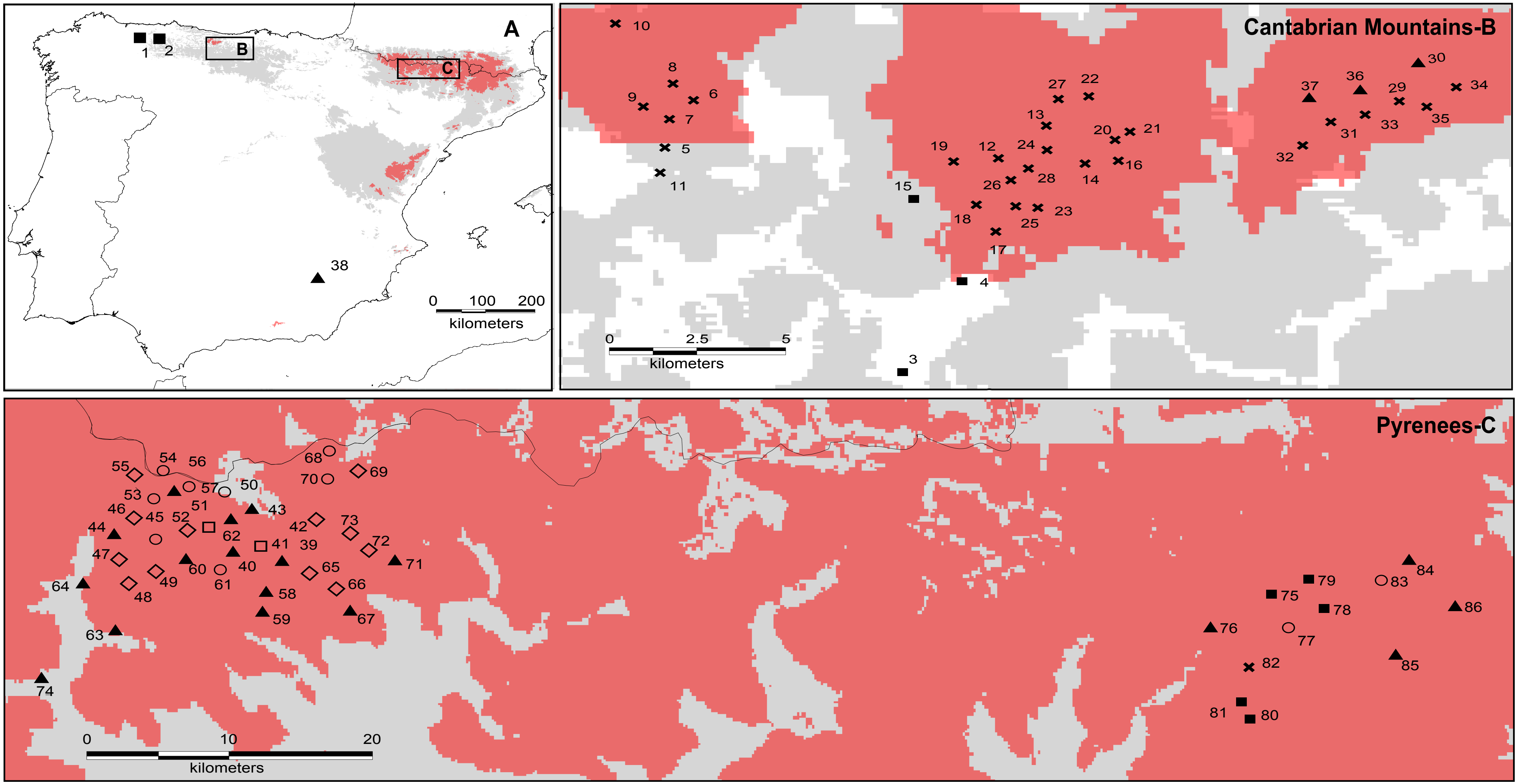

Figure S3. Ecological niche models of *F. eskia* (1) and *F. gautieri* (2) projected to LGM (grey) and to current (green for *F. eskia* and red for *F. gautieri*) climatic conditions. Predominance of one color indicates niche differentiation while dark color indicates overlapping of niche models. Maps were generated with Idrisi Selva v.17.02 environment (Clark Labs, Clark University, www.clarklabs.org).

Past climate changes facilitated homoploid speciation in three mountain spiny fescues (Festuca, Poaceae)
Marques I, Draper D, López-Herranz ML, Garnatje T, Segarra-Moragues JG, Catalán P.

1)

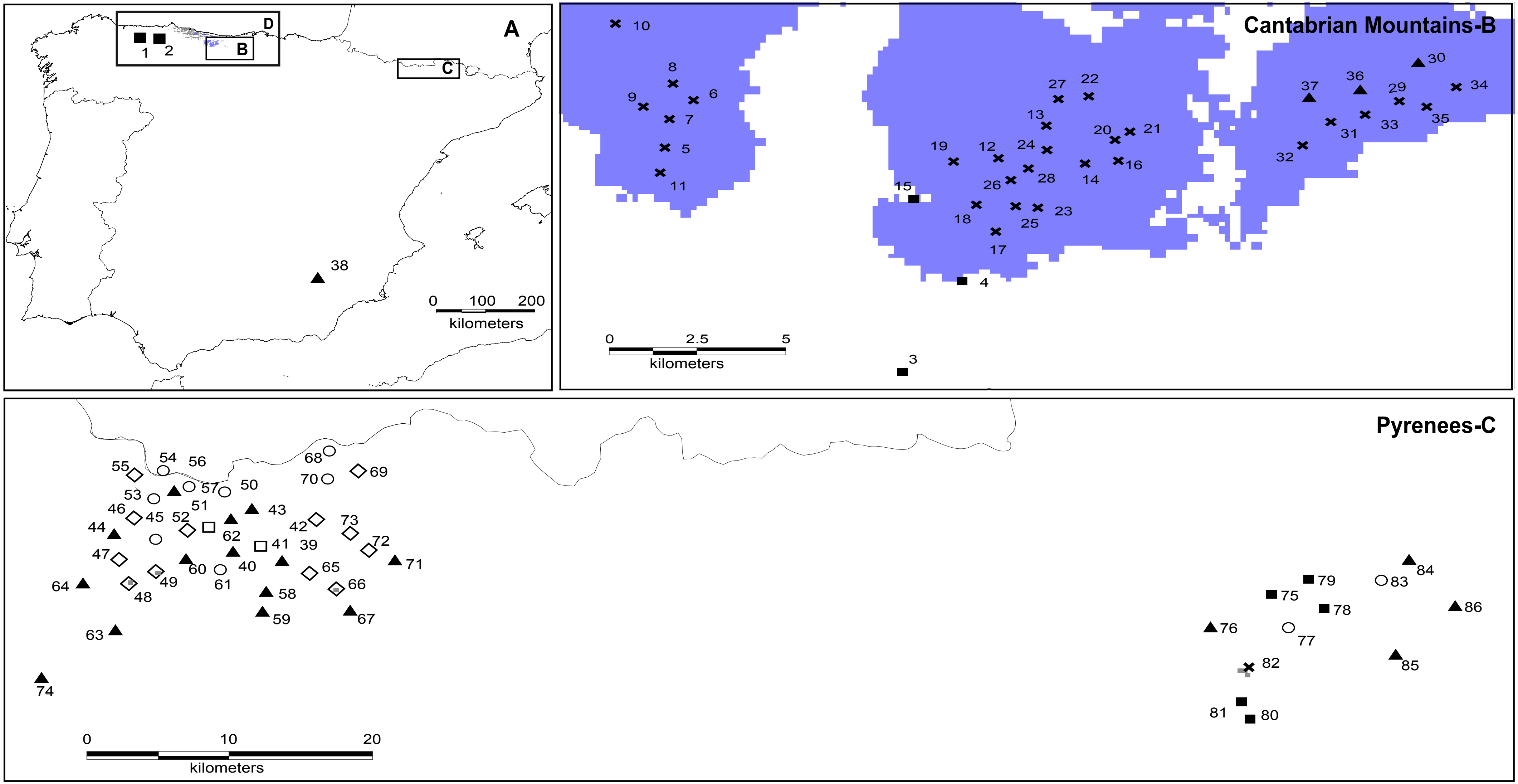

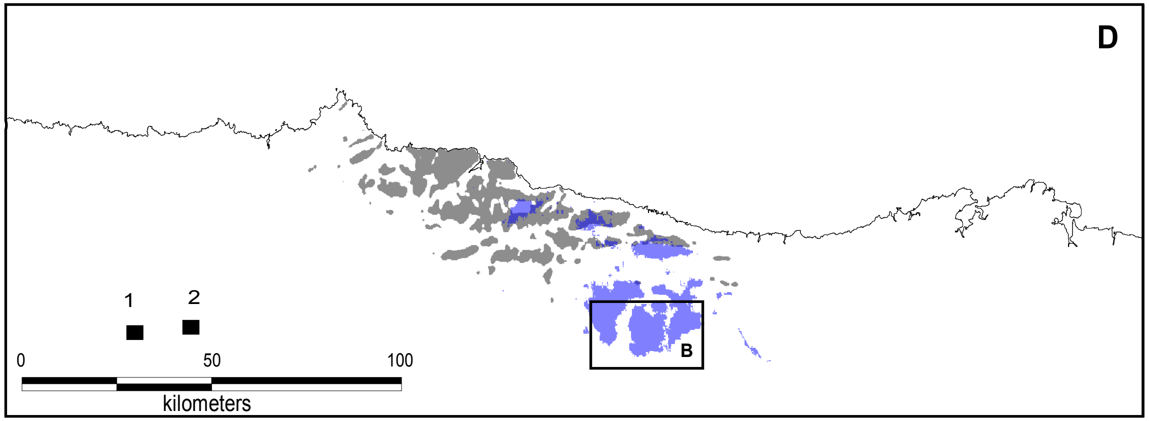

Figure S4. 1- Ecological niche models under current climate (blue) and LGM-CCSM (grey) scenarios across the whole Iberian area, based on the Maxent algorithm using only the Pyrenean records of *F. picoeuropeana*. B, D) Projections in the Cantabrian Mountains; C) Projections in the Pyrenees. Maps were generated with Idrisi Selva v.17.02 environment (Clark Labs, Clark University, www.clarklabs.org).

Past climate changes facilitated homoploid speciation in three mountain spiny fescues (Festuca, Poaceae)
Marques I, Draper D, López-Herranz ML, Garnatje T, Segarra-Moragues JG, Catalán P.

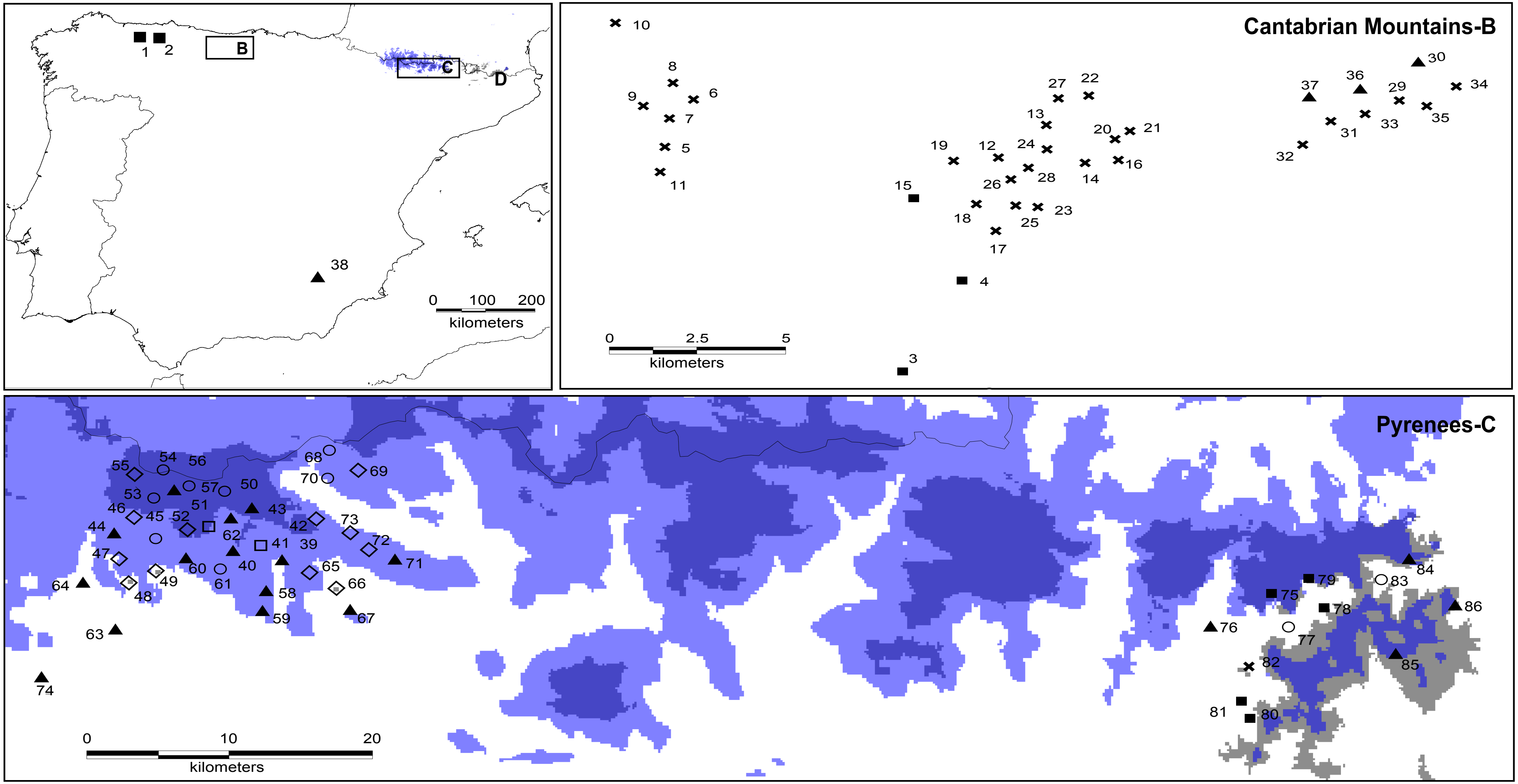

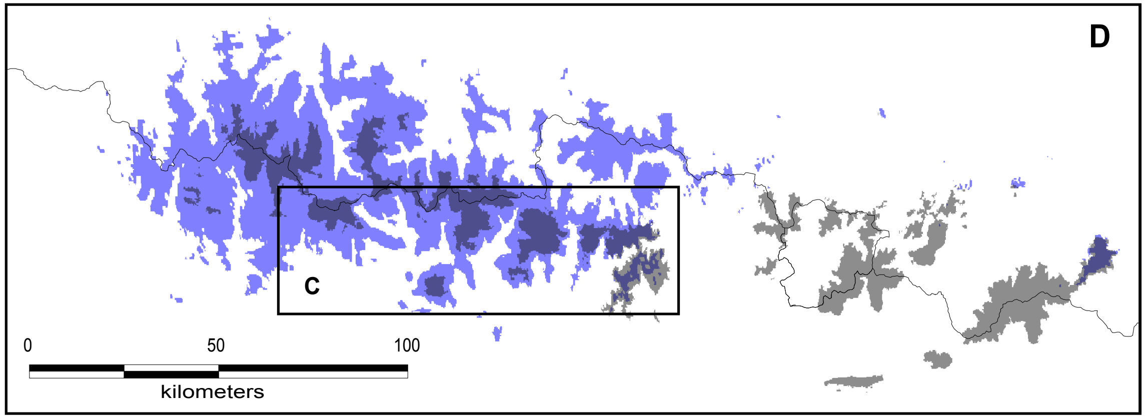

Figure S4. 1- 2- Ecological niche models under current climate (blue) and LGM-CCSM (grey) scenarios across the whole Iberian area, based on the Maxent algorithm using only the Cantabrian records of *F. picoeuropeana*. B) Projections in the Cantabrian Mountains; C, D) Projections in the Pyrenees. Maps were generated with Idrisi Selva v.17.02 environment (Clark Labs, Clark University, www.clarklabs.org).

Past climate changes facilitated homoploid speciation in three mountain spiny fescues (Festuca, Poaceae)

Marques I, Draper D, López-Herranz ML, Garnatje T, Segarra-Moragues JG, Catalán P.

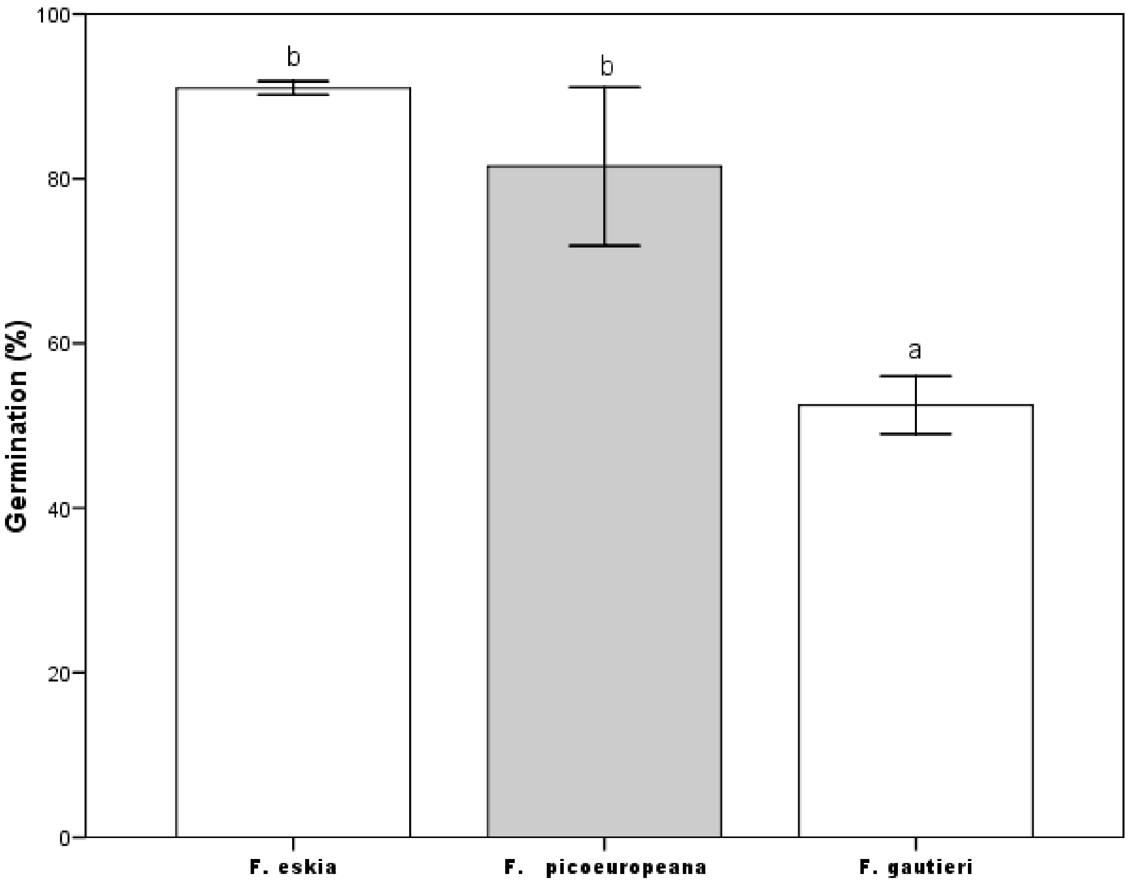

Figure S5. Percentage of seed germination of *Festuca eskia*, *F. gautieri* and *F. picoeuropeana*.

Past climate changes facilitated homoploid speciation in three mountain spiny fescues (Festuca, Poaceae)
Marques I, Draper D, López-Herranz ML, Garnatje T, Segarra-Moragues JG, Catalán P.
