# Supplementary material for: Past climate changes facilitated homoploid speciation in three mountain spiny fescues (Festuca, Poaceae)
Source: Sci Rep. 2016 Nov 3;6:36283. doi: 10.1038/srep36283 (PMC5093761; doi:10.1038/srep36283)
Supplement: Supplementary Table S4 [file srep36283-s5.doc]

**Past climate changes facilitated homoploid speciation in three mountain spiny fescues (Festuca, Poaceae)**

**Marques I, Draper D, López-Herranz ML, Garnatje T, Segarra-Moragues JG, Catalán P.**

**Table S4.** Current and LGM spatial niche overlap values between the parental species and the two subpopulations of *F. picoeuropeana*. Niche overlap values are based on Schoener’s *D* metric and Hellinger’s-based *I*. Pairwise background tests comparisons were evaluated by climate conditions only. Overlap values smaller that the null distribution support niche divergence (D) and values larger than it niche conservatism (C); when niche overlap values are similar to the null distribution background the results are inconclusive (NC). Significance: ** *P*<0.01; *P<0.05.

| **Species Pair (Sp1-Sp2)** |  |  | Background test | |
| --- | --- | --- | --- | --- |
|  | **Schoener's *D*** | ***I* Statistic** | **Sp1-Sp2** | **Sp2-Sp1** |
| **Present climate** |  |  |  |  |
| *F. eskia* - *F. gautieri* | 0.2186 | 0.8346^**^ | 0.2734^*^D | 0.3651^*^D |
| *F. eskia* - *F. picoeuropeana* (Cantabrian Mountains) | 0.2347 | 0.6939^**^ | 0.3215^*^D | 0.3982^*^D |
| *F. eskia* - *F. picoeuropeana* (Pyrenees) | 0.2143 | 0.8762^**^ | 0.1281^*^C | 0.1967^*^C |
| *F. gautieri* - *F. picoeuropeana* (Cantabrian Mountains) | 0.1213 | 0.5943^*^ | 0.2287^*^D | 0.1876^*^D |
| *F. gautieri* - *F. picoeuropeana* (Pyrenees) | 0.2136 | 0.7623^**^ | 0.1264^*^C | 0.1765^*^C |
| *F. picoeuropeana* (Cantabrian Mountains) - *F. picoeuropeana* (Pyrenees) | 0.0721 | 0.6892^**^ | 0.2913^*^D | 0.2236^*^D |
| **LGM** |  |  |  |  |
| *F. eskia* - *F. gautieri* | 0.4972 | 0.8384^**^ | _ | _ |
| *F. eskia* - *F. picoeuropeana* (Cantabrian Mountains) | 0.3819 | 0.7485^**^ | _ | _ |
| *F. eskia* - *F. picoeuropeana* (Pyrenees) | 0.3217 | 0.6945^**^ | _ | _ |
| *F. gautieri* - *F. picoeuropeana* (Cantabrian Mountains) | 0.2968 | 0.8452^*^ | _ | _ |
| *F. gautieri* - *F. picoeuropeana* (Pyrenees) | 0.3019 | 0.8842^**^ | _ | _ |
| *F. picoeuropeana* (Cantabrian Mountains) - *F. picoeuropeana* (Pyrenees) | 0.3461 | 0.6369^**^ | _ | _ |
