# Supplementary material for: Past climate changes facilitated homoploid speciation in three mountain spiny fescues (Festuca, Poaceae)
Source: Sci Rep. 2016 Nov 3;6:36283. doi: 10.1038/srep36283 (PMC5093761; doi:10.1038/srep36283)
Supplement: Supplementary Table S6 [file srep36283-s7.doc]

**Past climate changes facilitated homoploid speciation in three mountain spiny fescues (Festuca, Poaceae)**

**Marques I, Draper D, López-Herranz ML, Garnatje T, Segarra-Moragues JG, Catalán P.**

**Table S6.** List of uncorrelated variables used to build ENMs and to test niche divergence.

|  |  |  |
| --- | --- | --- |
| **Variable** | **Code** | **Data Source** |
| Altitude | Alt | srtm.csi.cgiar.org |
| Annual Mean Temperature | BIO1 | worldclim.org |
| Mean Diurnal Range | BIO2 | worldclim.org |
| Isothermality | BIO3 | worldclim.org |
| Temperature Seasonality | BIO4 | worldclim.org |
| Mean Temperature of Warmest Quarter | BIO10 | worldclim.org |
| Annual Precipitation | BIO12 | worldclim.org |
